# (η^5^-Cyclo­penta­dien­yl)(η^6^-1,2-dipyrrolidin-1-ylbenzene)iron(II) hexa­fluoridophosphate

**DOI:** 10.1107/S1600536809028116

**Published:** 2009-07-22

**Authors:** Hilary A. Jenkins, Jason D. Masuda, Adam Piórko

**Affiliations:** a Department of Chemistry, Saint Mary’s University, Halifax, Nova Scotia, Canada B3H 3C3

## Abstract

Both complexed rings in the iron(II) complex cation of the title salt, [Fe(C_5_H_5_)(C_14_H_20_N_2_)]PF_6_, are almost parallel [dihedral angle between planes = 5.34 (13)°]. Among the C atoms of the complexed arene ring, the quaternary C atoms are located at the longest, albeit unequal, distances from the Fe atom [2.252 (2) and 2.168 (2) Å].

## Related literature

For the synthesis of the title compound and related structures, see: Lee *et al.* (1989 ▶). For the crystal structures of {(η^5^-Cp)(η^6^-arene) Fe(II)}^+^ salts, see: Nesmeyanov *et al.* (1977 ▶); Dubois *et al.* (1989 ▶); Piórko *et al.* (1995 ▶); Manzur *et al.* (2000 ▶); Fuente­alba *et al.* (2007 ▶); Manzur *et al.* (2009 ▶) and literature cited therein.
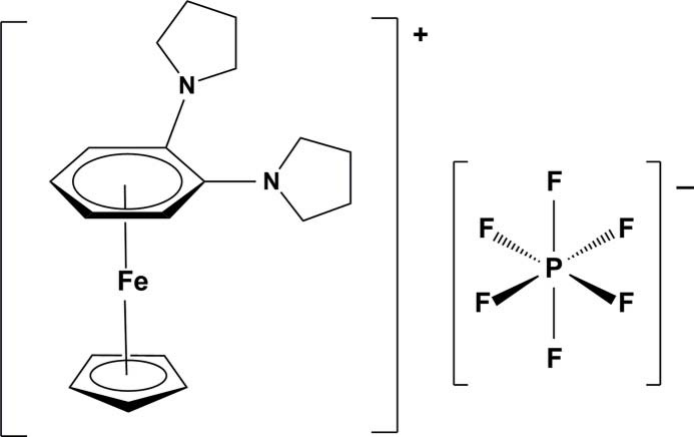


## Experimental

### 

#### Crystal data

[Fe(C_5_H_5_)(C_14_H_20_N_2_)]PF_6_
*M* *_r_* = 482.23
Triclinic, 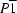

*a* = 9.7303 (6) Å
*b* = 10.6021 (6) Å
*c* = 10.6548 (6) Å
α = 93.845 (1)°
β = 113.259 (1)°
γ = 95.822 (1)°
*V* = 997.7 (1) Å^3^
*Z* = 2
Mo *K*α radiation
μ = 0.90 mm^−1^
*T* = 223 K
0.23 × 0.20 × 0.16 mm

#### Data collection

Bruker SMART CCD area-detector diffractometer
Absorption correction: multi-scan (*SADABS*; Bruker, 1998 ▶) *T*_min_ = 0.742, *T*_max_ = 0.866
7374 measured reflections
3501 independent reflections
2493 reflections with *I* > 2σ(*I*)
*R*_int_ = 0.017

#### Refinement

*R*[*F* ^2^ > 2σ(*F* ^2^)] = 0.033
*wR*(*F* ^2^) = 0.080
*S* = 0.92
3501 reflections
262 parameters
H-atom parameters constrained
Δρ_max_ = 0.30 e Å^−3^
Δρ_min_ = −0.21 e Å^−3^

### 

Data collection: *SMART* (Bruker, 1998 ▶); cell refinement: *SAINT-Plus* (Bruker, 1998 ▶); data reduction: *SAINT-Plus*; program(s) used to solve structure: *SHELXS97* (Sheldrick, 2008 ▶); program(s) used to refine structure: *SHELXL97* (Sheldrick, 2008 ▶); molecular graphics: *ORTEP-3 for Windows* (Farrugia, 1997 ▶); software used to prepare material for publication: *SHELXL97*.

## Supplementary Material

Crystal structure: contains datablocks I, global. DOI: 10.1107/S1600536809028116/si2188sup1.cif

Structure factors: contains datablocks I. DOI: 10.1107/S1600536809028116/si2188Isup2.hkl

Additional supplementary materials:  crystallographic information; 3D view; checkCIF report

## Figures and Tables

**Fig. 1.**
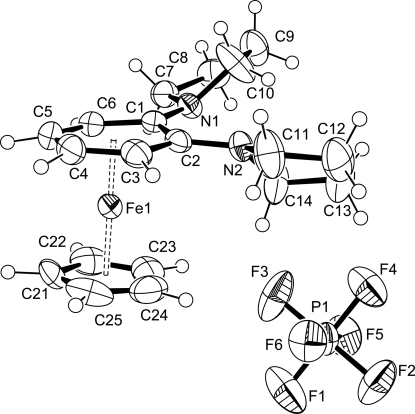
View of the cation showing the labelling of non-H atoms with the thermal ellipsoids shown at 50% probability levels.

## supplementary crystallographic information

### Comment

The title compound, along with similar mono- and/or di-*N*-butylamino- and cyano-arenes complexed with a cyclopentadienyliron(II) moiety, were reported in the study of nucleophilic aromatic mono- and di-substitution reactions using 1,2-, 1,3-, and 1,4-dichlorobenzene FeCp complexes (Lee *et al.*, 1989). The *ORTEP* of the title compound is shown in Figure 1. The two aromatic rings are not planar, with an angle of 5.34 (13)° between the planes formed by C1···C6 and C21···C25. This is the largest value among those reported previously by our group, although lower than the values 7° reported for 1,1'-trimethylenebenzene-CpFe cation (Nesmeyanov *et al.*, 1977), and 5.4° reported for hexaethylbenzene-CpFe complex (Dubois *et al.*, 1989). No standard uncertainties were given by these authors.

The Fe ion is located at the distances 1.6601 (12) Å from the Cp and 1.5680 (11) Å from the phenyl ring, and these values are close to those reported in the literature for similar complexes (see for example Piórko *et al.*, 1995; Fuentealba *et al.*, 2007; Manzur *et al.*, 2009; and literature cited therein). The Fe - C1 distance at 2.252 (2) Å (where C1 is one of the quaternary carbon atoms in the phenyl ring bonding N1 of pyrrolidinyl substituent), is the longest among the distances Fe to C atoms of this ring. The distance Fe - C2 (where C2 is another quaternary carbon atom of the complexed phenyl ring) is significantly shorter at 2.168 (2) Å. While this second value is typical for an aromatic C atom (in a FeCp or Fe-pentamethylCp complexed phenyl ring) to Fe distance (see for example Piórko *et al.*, 1995; Manzur *et al.*, 2000; Fuentealba *et al.*, 2007; Manzur *et al.*, 2009, and literature cited therein), the Fe - C1 distance is the longest one reported, to the best of our knowledge, for similar iron complexes. In a complexed arene ring, the longest bond between carbon atoms of this ring is found for C1 - C2 quaternary carbon atoms at 1.444 (3) Å, while all other similar bonds are found in the range 1.398 (4) to 1.427 (3) Å. Both nitrogen atoms show similar bond lengths toward aromatic ring carbon atoms [1.377 (3) Å for a ring N1 - C7···C10 and 1.386 (3) Å for a ring N2 - C11···C14], and toward methylene carbon atoms of pyrrolidinyl rings [range 1.453 (3) to 1.467 (3) Å]. One of the pyrrolidinyl rings (N2 - C11···C14) appears to be quite symmetrical and located in and below the plane of the metal-complexed phenyl ring, while another one (N1 - C7···C10) shows distorted both interatomic distances and angles, and is located above the plane of the complexed phenyl ring. The planes defined by the carbon atoms of both pyrrolidinyl rings are tilted with respect to the phenyl ring plane by 32.12(0.17)° for a plane C11···C14 and 29.40(0.13)° for the plane C7···C10.

### Experimental

The title compound was prepared following the method of Lee *et al.* (1989). A crystal used for data collection was grown by slow evaporation of solvents from a solution of the complex in acetone-diethyl ether-dichloromethane mixture at 280 K.

### Refinement

The H atoms were placed in geometrically idealized positions with C-H distances of 0.99Å (aromatic) and 0.98Å (methylene) and constrained to ride on the parent C atom with Uiso(H) = 1.2Ueq(C) for aromatic and methylene protons.

### Crystal data

**Table:**

[Fe(C_5_H_5_)(C_14_H_20_N_2_)]PF_6_	*Z* = 2
*M**_r_* = 482.23	*F*(000) = 496
Triclinic, *P*1	*D*_x_ = 1.605 Mg m^−^^3^
Hall symbol: -P 1	Mo *K*α radiation, λ = 0.71073 Å
*a* = 9.7303 (6) Å	Cell parameters from 2668 reflections
*b* = 10.6021 (6) Å	θ = 23.7–2.8°
*c* = 10.6548 (6) Å	µ = 0.90 mm^−^^1^
α = 93.845 (1)°	*T* = 223 K
β = 113.259 (1)°	Block, orange
γ = 95.822 (1)°	0.23 × 0.20 × 0.16 mm
*V* = 997.7 (1) Å^3^	

### Data collection

**Table:**

Bruker SMART CCD area-detector diffractometer	3501 independent reflections
Radiation source: fine-focus sealed tube	2493 reflections with *I* > 2σ(*I*)
graphite	*R*_int_ = 0.017
φ and ω scans	θ_max_ = 25.0°, θ_min_ = 1.9°
Absorption correction: multi-scan (*SADABS*; Bruker, 1998)	*h* = −11→11
*T*_min_ = 0.742, *T*_max_ = 0.866	*k* = −12→12
7374 measured reflections	*l* = −12→12

### Refinement

**Table:**

Refinement on *F*^2^	Primary atom site location: structure-invariant direct methods
Least-squares matrix: full	Secondary atom site location: difference Fourier map
*R*[*F*^2^ > 2σ(*F*^2^)] = 0.033	Hydrogen site location: inferred from neighbouring sites
*wR*(*F*^2^) = 0.080	H-atom parameters constrained
*S* = 0.92	*w* = 1/[σ^2^(*F*_o_^2^) + (0.0452*P*)^2^] where *P* = (*F*_o_^2^ + 2*F*_c_^2^)/3
3501 reflections	(Δ/σ)_max_ = 0.001
262 parameters	Δρ_max_ = 0.30 e Å^−^^3^
0 restraints	Δρ_min_ = −0.21 e Å^−^^3^

### Special details

**Table:**

Geometry. All e.s.d.'s (except the e.s.d. in the dihedral angle between two l.s. planes) are estimated using the full covariance matrix. The cell e.s.d.'s are taken into account individually in the estimation of e.s.d.'s in distances, angles and torsion angles; correlations between e.s.d.'s in cell parameters are only used when they are defined by crystal symmetry. An approximate (isotropic) treatment of cell e.s.d.'s is used for estimating e.s.d.'s involving l.s. planes.
Refinement. Refinement of *F*^2^ against ALL reflections. The weighted *R*-factor *wR* and goodness of fit *S* are based on *F*^2^, conventional *R*-factors *R* are based on *F*, with *F* set to zero for negative *F*^2^. The threshold expression of *F*^2^ > σ(*F*^2^) is used only for calculating *R*-factors(gt) *etc*. and is not relevant to the choice of reflections for refinement. *R*-factors based on *F*^2^ are statistically about twice as large as those based on *F*, and *R*- factors based on ALL data will be even larger.

### Fractional atomic coordinates and isotropic or equivalent isotropic displacement parameters (Å^2^)

**Table:**

	*x*	*y*	*z*	*U*_iso_*/*U*_eq_	
Fe1	0.67408 (4)	0.24243 (4)	0.77597 (4)	0.03565 (13)	
N1	0.3474 (2)	0.07175 (19)	0.7496 (2)	0.0355 (5)	
N2	0.3557 (2)	0.34299 (19)	0.7376 (2)	0.0369 (5)	
C1	0.4793 (3)	0.1542 (2)	0.8193 (2)	0.0352 (6)	
C2	0.4827 (3)	0.2905 (2)	0.8161 (2)	0.0346 (6)	
C3	0.6194 (3)	0.3704 (3)	0.8986 (3)	0.0434 (7)	
H3A	0.6279	0.4618	0.8844	0.052*	
C4	0.7521 (3)	0.3211 (3)	0.9780 (3)	0.0512 (8)	
H4A	0.8495	0.3787	1.0197	0.061*	
C5	0.7520 (3)	0.1888 (3)	0.9703 (3)	0.0493 (7)	
H5A	0.8491	0.1539	1.0073	0.059*	
C6	0.6191 (3)	0.1074 (3)	0.8890 (3)	0.0425 (7)	
H6A	0.6259	0.0167	0.8668	0.051*	
C7	0.3512 (3)	−0.0664 (2)	0.7431 (3)	0.0454 (7)	
H7A	0.3983	−0.0926	0.8354	0.055*	
H7B	0.4065	−0.0930	0.6886	0.055*	
C8	0.1851 (3)	−0.1220 (3)	0.6735 (3)	0.0562 (8)	
H8A	0.1530	−0.1386	0.5737	0.067*	
H8B	0.1661	−0.2020	0.7087	0.067*	
C9	0.1023 (3)	−0.0212 (3)	0.7086 (4)	0.0620 (9)	
H9A	0.0186	−0.0044	0.6254	0.074*	
H9B	0.0612	−0.0498	0.7739	0.074*	
C10	0.2115 (3)	0.0940 (3)	0.7695 (4)	0.0764 (11)	
H10A	0.2345	0.1108	0.8677	0.092*	
H10B	0.1710	0.1678	0.7244	0.092*	
C11	0.3514 (3)	0.4792 (3)	0.7581 (4)	0.0679 (10)	
H11A	0.4326	0.5284	0.7420	0.081*	
H11B	0.3617	0.5053	0.8517	0.081*	
C12	0.2003 (4)	0.4983 (3)	0.6547 (4)	0.0713 (10)	
H12A	0.1281	0.5017	0.6976	0.086*	
H12B	0.2075	0.5782	0.6149	0.086*	
C13	0.1514 (4)	0.3865 (3)	0.5463 (4)	0.0756 (11)	
H13A	0.1370	0.4149	0.4569	0.091*	
H13B	0.0561	0.3390	0.5387	0.091*	
C14	0.2751 (3)	0.3043 (3)	0.5904 (3)	0.0631 (9)	
H14A	0.2333	0.2137	0.5718	0.076*	
H14B	0.3419	0.3198	0.5425	0.076*	
C21	0.8544 (3)	0.2220 (4)	0.7290 (3)	0.0636 (9)	
H21A	0.9544	0.2032	0.7924	0.076*	
C22	0.7286 (4)	0.1314 (3)	0.6439 (3)	0.0589 (9)	
H22A	0.7238	0.0373	0.6360	0.071*	
C23	0.6132 (4)	0.2020 (4)	0.5693 (3)	0.0625 (9)	
H23A	0.5112	0.1653	0.5009	0.075*	
C24	0.6654 (5)	0.3286 (4)	0.6074 (4)	0.0705 (10)	
H24A	0.6073	0.3993	0.5715	0.085*	
C25	0.8119 (5)	0.3425 (3)	0.7035 (4)	0.0712 (10)	
H25A	0.8767	0.4249	0.7479	0.085*	
P1	0.22457 (8)	0.25827 (7)	0.16756 (8)	0.0460 (2)	
F1	0.3570 (2)	0.2424 (2)	0.1210 (2)	0.1058 (8)	
F2	0.1610 (2)	0.35070 (18)	0.0551 (2)	0.0988 (7)	
F3	0.2889 (2)	0.16555 (17)	0.2805 (2)	0.1010 (8)	
F4	0.0910 (2)	0.27354 (19)	0.2133 (2)	0.0860 (6)	
F5	0.12317 (19)	0.14086 (16)	0.05954 (19)	0.0766 (6)	
F6	0.32525 (19)	0.37579 (15)	0.27729 (18)	0.0713 (5)	

### Atomic displacement parameters (Å^2^)

**Table:**

	*U*^11^	*U*^22^	*U*^33^	*U*^12^	*U*^13^	*U*^23^
Fe1	0.0302 (2)	0.0442 (3)	0.0345 (2)	0.00790 (17)	0.01440 (17)	0.00601 (17)
N1	0.0326 (12)	0.0336 (12)	0.0445 (13)	0.0067 (10)	0.0194 (11)	0.0058 (10)
N2	0.0322 (12)	0.0343 (13)	0.0428 (13)	0.0092 (10)	0.0131 (10)	0.0007 (10)
C1	0.0345 (15)	0.0473 (17)	0.0299 (14)	0.0096 (13)	0.0180 (12)	0.0070 (12)
C2	0.0329 (15)	0.0423 (16)	0.0321 (14)	0.0044 (12)	0.0177 (12)	0.0008 (12)
C3	0.0415 (17)	0.0507 (17)	0.0375 (15)	0.0010 (14)	0.0185 (14)	−0.0051 (13)
C4	0.0342 (16)	0.080 (2)	0.0318 (16)	−0.0004 (16)	0.0086 (13)	−0.0041 (15)
C5	0.0366 (17)	0.075 (2)	0.0382 (16)	0.0164 (16)	0.0132 (14)	0.0172 (16)
C6	0.0389 (16)	0.0540 (18)	0.0416 (16)	0.0140 (14)	0.0203 (14)	0.0163 (14)
C7	0.0524 (18)	0.0412 (17)	0.0498 (17)	0.0138 (14)	0.0257 (15)	0.0100 (14)
C8	0.060 (2)	0.0476 (19)	0.060 (2)	−0.0034 (16)	0.0261 (17)	0.0046 (16)
C9	0.0458 (18)	0.0486 (19)	0.093 (3)	0.0076 (16)	0.0270 (18)	0.0188 (18)
C10	0.0490 (19)	0.060 (2)	0.132 (3)	−0.0054 (17)	0.056 (2)	−0.015 (2)
C11	0.052 (2)	0.049 (2)	0.088 (3)	0.0189 (16)	0.0138 (19)	−0.0108 (18)
C12	0.065 (2)	0.056 (2)	0.097 (3)	0.0278 (18)	0.031 (2)	0.015 (2)
C13	0.067 (2)	0.067 (2)	0.074 (2)	0.0283 (19)	0.0037 (19)	0.009 (2)
C14	0.054 (2)	0.071 (2)	0.0473 (19)	0.0312 (17)	−0.0009 (16)	−0.0007 (17)
C21	0.0356 (18)	0.113 (3)	0.056 (2)	0.0284 (19)	0.0284 (16)	0.017 (2)
C22	0.087 (3)	0.0439 (19)	0.071 (2)	0.0201 (18)	0.056 (2)	0.0065 (17)
C23	0.053 (2)	0.101 (3)	0.0357 (17)	0.009 (2)	0.0230 (16)	−0.0054 (18)
C24	0.093 (3)	0.080 (3)	0.072 (2)	0.039 (2)	0.058 (2)	0.035 (2)
C25	0.086 (3)	0.061 (2)	0.090 (3)	−0.009 (2)	0.067 (2)	−0.002 (2)
P1	0.0372 (4)	0.0382 (4)	0.0523 (5)	0.0053 (3)	0.0078 (4)	0.0025 (4)
F1	0.0534 (12)	0.1385 (19)	0.1172 (18)	−0.0023 (12)	0.0375 (12)	−0.0366 (15)
F2	0.1071 (17)	0.0761 (14)	0.0849 (15)	0.0096 (12)	0.0053 (13)	0.0378 (12)
F3	0.1097 (17)	0.0581 (12)	0.0898 (15)	0.0137 (11)	−0.0105 (13)	0.0263 (11)
F4	0.0559 (12)	0.1074 (16)	0.0916 (15)	−0.0012 (11)	0.0342 (11)	−0.0184 (12)
F5	0.0623 (12)	0.0644 (12)	0.0777 (13)	−0.0119 (9)	0.0115 (10)	−0.0220 (10)
F6	0.0672 (12)	0.0488 (10)	0.0784 (13)	−0.0098 (9)	0.0158 (10)	−0.0095 (9)

### Geometric parameters (Å, °)

**Table:**

Fe1—C22	2.035 (3)	C9—C10	1.459 (4)
Fe1—C21	2.032 (3)	C9—H9A	0.9800
Fe1—C25	2.039 (3)	C9—H9B	0.9800
Fe1—C23	2.044 (3)	C10—H10A	0.9800
Fe1—C24	2.046 (3)	C10—H10B	0.9800
Fe1—C5	2.047 (3)	C11—C12	1.492 (4)
Fe1—C4	2.061 (3)	C11—H11A	0.9800
Fe1—C3	2.071 (3)	C11—H11B	0.9800
Fe1—C6	2.090 (2)	C12—C13	1.495 (4)
Fe1—C2	2.168 (2)	C12—H12A	0.9800
Fe1—C1	2.252 (2)	C12—H12B	0.9800
N1—C1	1.377 (3)	C13—C14	1.500 (4)
N1—C10	1.458 (3)	C13—H13A	0.9800
N1—C7	1.467 (3)	C13—H13B	0.9800
N2—C2	1.386 (3)	C14—H14A	0.9800
N2—C11	1.453 (3)	C14—H14B	0.9800
N2—C14	1.456 (3)	C21—C25	1.394 (4)
C1—C6	1.427 (3)	C21—C22	1.418 (4)
C1—C2	1.444 (3)	C21—H21A	0.9900
C2—C3	1.417 (3)	C22—C23	1.414 (4)
C3—C4	1.411 (4)	C22—H22A	0.9900
C3—H3A	0.9900	C23—C24	1.361 (4)
C4—C5	1.400 (4)	C23—H23A	0.9900
C4—H4A	0.9900	C24—C25	1.374 (5)
C5—C6	1.398 (4)	C24—H24A	0.9900
C5—H5A	0.9900	C25—H25A	0.9900
C6—H6A	0.9900	P1—F1	1.572 (2)
C7—C8	1.520 (4)	P1—F2	1.5731 (19)
C7—H7A	0.9800	P1—F4	1.5762 (19)
C7—H7B	0.9800	P1—F3	1.5791 (19)
C8—C9	1.514 (4)	P1—F5	1.5841 (17)
C8—H8A	0.9800	P1—F6	1.5910 (17)
C8—H8B	0.9800		
			
C22—Fe1—C21	40.80 (12)	C1—C6—H6A	118.5
C22—Fe1—C25	67.36 (12)	Fe1—C6—H6A	118.5
C21—Fe1—C25	40.05 (13)	N1—C7—C8	103.2 (2)
C22—Fe1—C23	40.55 (12)	N1—C7—H7A	111.1
C21—Fe1—C23	67.63 (12)	C8—C7—H7A	111.1
C25—Fe1—C23	66.04 (14)	N1—C7—H7B	111.1
C22—Fe1—C24	67.07 (13)	C8—C7—H7B	111.1
C21—Fe1—C24	67.07 (13)	H7A—C7—H7B	109.1
C25—Fe1—C24	39.29 (13)	C9—C8—C7	104.9 (2)
C23—Fe1—C24	38.88 (12)	C9—C8—H8A	110.8
C22—Fe1—C5	112.92 (13)	C7—C8—H8A	110.8
C21—Fe1—C5	100.20 (12)	C9—C8—H8B	110.8
C25—Fe1—C5	122.30 (15)	C7—C8—H8B	110.8
C23—Fe1—C5	150.64 (14)	H8A—C8—H8B	108.8
C24—Fe1—C5	161.28 (15)	C10—C9—C8	107.1 (2)
C22—Fe1—C4	142.85 (13)	C10—C9—H9A	110.3
C21—Fe1—C4	108.64 (12)	C8—C9—H9A	110.3
C25—Fe1—C4	103.36 (13)	C10—C9—H9B	110.3
C23—Fe1—C4	167.81 (14)	C8—C9—H9B	110.3
C24—Fe1—C4	128.99 (15)	H9A—C9—H9B	108.6
C5—Fe1—C4	39.85 (11)	C9—C10—N1	106.9 (2)
C22—Fe1—C3	174.17 (12)	C9—C10—H10A	110.3
C21—Fe1—C3	137.04 (13)	N1—C10—H10A	110.3
C25—Fe1—C3	107.72 (12)	C9—C10—H10B	110.3
C23—Fe1—C3	135.23 (13)	N1—C10—H10B	110.3
C24—Fe1—C3	107.17 (13)	H10A—C10—H10B	108.6
C5—Fe1—C3	72.16 (11)	N2—C11—C12	104.5 (2)
C4—Fe1—C3	39.95 (10)	N2—C11—H11A	110.9
C22—Fe1—C6	101.81 (11)	C12—C11—H11A	110.9
C21—Fe1—C6	117.43 (12)	N2—C11—H11B	110.9
C25—Fe1—C6	155.33 (14)	C12—C11—H11B	110.9
C23—Fe1—C6	120.86 (13)	H11A—C11—H11B	108.9
C24—Fe1—C6	158.35 (15)	C13—C12—C11	106.0 (2)
C5—Fe1—C6	39.48 (10)	C13—C12—H12A	110.5
C4—Fe1—C6	71.30 (11)	C11—C12—H12A	110.5
C3—Fe1—C6	83.94 (11)	C13—C12—H12B	110.5
C22—Fe1—C2	142.09 (12)	C11—C12—H12B	110.5
C21—Fe1—C2	171.95 (12)	H12A—C12—H12B	108.7
C25—Fe1—C2	131.90 (13)	C12—C13—C14	106.3 (3)
C23—Fe1—C2	110.32 (11)	C12—C13—H13A	110.5
C24—Fe1—C2	106.23 (12)	C14—C13—H13A	110.5
C5—Fe1—C2	85.14 (10)	C12—C13—H13B	110.5
C4—Fe1—C2	71.66 (10)	C14—C13—H13B	110.5
C3—Fe1—C2	38.97 (9)	H13A—C13—H13B	108.7
C6—Fe1—C2	70.48 (10)	N2—C14—C13	104.1 (2)
C22—Fe1—C1	114.95 (11)	N2—C14—H14A	110.9
C21—Fe1—C1	149.61 (13)	C13—C14—H14A	110.9
C25—Fe1—C1	166.39 (14)	N2—C14—H14B	110.9
C23—Fe1—C1	106.37 (11)	C13—C14—H14B	110.9
C24—Fe1—C1	127.86 (14)	H14A—C14—H14B	109.0
C5—Fe1—C1	70.06 (10)	C25—C21—C22	106.9 (3)
C4—Fe1—C1	82.71 (10)	C25—C21—Fe1	70.24 (17)
C3—Fe1—C1	69.04 (10)	C22—C21—Fe1	69.70 (16)
C6—Fe1—C1	38.16 (9)	C25—C21—H21A	126.5
C2—Fe1—C1	38.09 (9)	C22—C21—H21A	126.5
C1—N1—C10	119.5 (2)	Fe1—C21—H21A	126.5
C1—N1—C7	119.5 (2)	C21—C22—C23	106.5 (3)
C10—N1—C7	107.0 (2)	C21—C22—Fe1	69.50 (16)
C2—N2—C11	120.5 (2)	C23—C22—Fe1	70.05 (16)
C2—N2—C14	121.6 (2)	C21—C22—H22A	126.7
C11—N2—C14	106.2 (2)	C23—C22—H22A	126.7
N1—C1—C6	121.1 (2)	Fe1—C22—H22A	126.7
N1—C1—C2	121.1 (2)	C24—C23—C22	108.7 (3)
C6—C1—C2	117.7 (2)	C24—C23—Fe1	70.64 (18)
N1—C1—Fe1	137.22 (17)	C22—C23—Fe1	69.40 (16)
C6—C1—Fe1	64.79 (13)	C24—C23—H23A	125.7
C2—C1—Fe1	67.82 (13)	C22—C23—H23A	125.7
N2—C2—C3	120.3 (2)	Fe1—C23—H23A	125.7
N2—C2—C1	121.6 (2)	C23—C24—C25	108.9 (3)
C3—C2—C1	118.1 (2)	C23—C24—Fe1	70.48 (18)
N2—C2—Fe1	131.77 (17)	C25—C24—Fe1	70.11 (19)
C3—C2—Fe1	66.82 (14)	C23—C24—H24A	125.5
C1—C2—Fe1	74.09 (14)	C25—C24—H24A	125.5
C4—C3—C2	122.3 (3)	Fe1—C24—H24A	125.5
C4—C3—Fe1	69.64 (15)	C24—C25—C21	109.0 (3)
C2—C3—Fe1	74.21 (14)	C24—C25—Fe1	70.60 (19)
C4—C3—H3A	118.2	C21—C25—Fe1	69.71 (17)
C2—C3—H3A	118.2	C24—C25—H25A	125.5
Fe1—C3—H3A	118.2	C21—C25—H25A	125.5
C5—C4—C3	119.2 (3)	Fe1—C25—H25A	125.5
C5—C4—Fe1	69.55 (16)	F1—P1—F2	89.96 (13)
C3—C4—Fe1	70.42 (15)	F1—P1—F4	179.60 (12)
C5—C4—H4A	119.5	F2—P1—F4	89.95 (12)
C3—C4—H4A	119.5	F1—P1—F3	89.87 (13)
Fe1—C4—H4A	119.5	F2—P1—F3	179.82 (14)
C6—C5—C4	119.7 (3)	F4—P1—F3	90.22 (12)
C6—C5—Fe1	71.92 (15)	F1—P1—F5	90.17 (11)
C4—C5—Fe1	70.60 (16)	F2—P1—F5	89.60 (11)
C6—C5—H5A	119.6	F4—P1—F5	89.45 (10)
C4—C5—H5A	119.6	F3—P1—F5	90.45 (10)
Fe1—C5—H5A	119.6	F1—P1—F6	90.50 (11)
C5—C6—C1	122.2 (3)	F2—P1—F6	90.75 (10)
C5—C6—Fe1	68.60 (15)	F4—P1—F6	89.89 (10)
C1—C6—Fe1	77.05 (15)	F3—P1—F6	89.20 (10)
C5—C6—H6A	118.5	F5—P1—F6	179.25 (12)
